# Vascular Cognitive Impairment (VCI) after non-embolic ischemic stroke during a 12-month follow-up in Brazil

**DOI:** 10.1590/S1980-57642012DN06030009

**Published:** 2012

**Authors:** Sonia Maria Dozzi Brucki, Michel Ferreira Machado, Maria Sheila Guimarães Rocha

**Affiliations:** MD, Hospital Santa Marcelina, São Paulo SP, Brazil.

**Keywords:** vascular cognitive impairment, cognitive impairment no dementia, vascular dementia, post-stroke dementia, post-stroke cognitive impairment

## Abstract

VCI represents a spectrum of cognitive impairments associated with stroke, vascular brain injury, or subclinical disease ranging from the least to most severe manifestations. Few studies are available on the prevalence of post-stroke VCI and none have been conducted in Brazil.

**Objective:**

To determine the prevalence rates of VCI and associated risk factors in a sample of ischemic stroke patients.

**Methods:**

We evaluated 172 patients with ischemic stroke for cognitive impairment one year after ictus.

**Results:**

Patients comprised 81 women (47.1%) and had a mean age of 67.77 (7.86) years, schooling of 3.52 (2.99) years, and MMSE score of 24.94 (3.59) points. After cognitive evaluation, 4.6% were diagnosed as CIND (cognitive impairment no dementia) and 12.2% had a diagnosis of dementia (probable vascular dementia in 20 patients and one subject with cerebrovascular disease and Alzheimer's disease).

**Conclusion:**

The prevalence of dementia was lower than previous reports but our sample had a lower age than others, while a 12 month-period of follow-up prevented interference from associated neurodegenerative disorders.

## Abstract (pt)

CCV representa um espectro de comprometimento cognitivo associado ao AVC, lesão cerebral vascular ou doença subclínica que vai desde as manifestações menos graves às mais graves. Há poucos estudos sobre a prevalência de CCV pós AVC e nenhum no Brasil.

**Objetivo:**

Determinar a prevalência de VCI em uma amostra de pacientes com AVC isquêmico e fatores de risco associados.

**Métodos:**

Foram avaliados 172 pacientes com AVC isquêmico após um ano do ictus, em relação ao comprometimento cognitivo.

**Resultados:**

Os pacientes foram compostos por 81 mulheres (47,1%) e tinham idade média de 67,77 (7,86) anos, a escolaridade de 3,52 (2,99) anos, a pontuação do MEEM de 24,94 (3,59) pontos. Após a avaliação cognitiva, 4,6% foram diagnosticados como CCSD (comprometimento cognitivo sem demência) e 12,2% tiveram diagnóstico de demência (demência vascular provável em 20 pacientes e um com doença cerebrovascular e doença de Alzheimer).

**CONCLUSÃO:**

A prevalência de demência na nossa população está entre as mais baixas, porém, nossos pacientes são mais jovens e foram acompanhados por um período de 12 meses, o que evita a contaminação por doenças neurodegenerativas associadas.

## INTRODUCTION

Vascular cognitive impairment (VCI) is a relatively new concept characterizing all possible cognitive levels of deficit presented after a stroke or with neuroimaging suggestive of cerebrovascular disease. VCI represents a spectrum of cognitive impairments associated with stroke, vascular brain injury, or subclinical disease ranging from the least to the most severe manifestations^1^.

The concept includes brains at risk for vascular cognitive impairment through to overt dementia and many criteria have been proposed differing in relation to severity, mixed pathologies, or prerequisite cognitive domains involved.^2-7^ The most recent criteria were published by Gorelick et al. who classified VCI into vascular mild cognitive impairment (VaMCI) and vascular dementia (VaD), based on functional impairment and number of evaluated cognitive domains, preferentially, with scores between 1 and 1.5 standard deviations from the expected average, corrected for schooling and age. The neuropsychological evaluation must include memory, visuospatial, language, and executive domains^1^.

Studies with the vascular dementia or VCI concepts^8^ show a greater prevalence with aging, and vascular dementia in epidemiological studies varies between 22 and 26.8%.^9,10^ Epidemiologic studies in Brazil reveal prevalences of 9.3% and 15.9% for vascular dementia (VaD) among demented participants^11,12^ whereas studies in tertiary outpatient clinics report a prevalence of vascular dementia between 24.9 and 32.25%,^13,14^ and of 36.9% in a sample with presenile dementia.^15^

When neuroimaging and pathological studies are considered, there are many variations regarding prevalence, with vascular lesions such as lacunes, hyperintensities of white matter, or pathological findings including lacunes, microinfarcts, demyelination, and microbleeds, common among studies.^16-20^ Currently, there is no consensus on neuropathologic criteria for vascular and mixed dementia.^21^ The main problem is the quantity and type of pathology observed in brain necessary to define impaired cognition.

Post-stroke dementia have been evaluated more frequently since the nineties, with great variability among studies with regard to length of follow-up, type of stroke, criteria for dementia, patient age, presence of previous dementia and so forth.

In a recent meta-analysis and systematic review, Pendlebury and Rothwell described post-stroke dementia rates from 7.4% in population-based studies of first-ever stroke, in which pre-stroke dementia was excluded, to 41.3% in hospital-based studies of recurrent stroke in which pre-stroke dementia was included. When all strokes (first and recurrent) including pre-stroke dementia were considered, the prevalence was 26.5%.^22^ In another review, the prevalence rates of post-stroke dementia ranged from 12.2% to 31.8% within three month to one year after stroke.^23^

The range of prevalence rates in the hospital-based stroke population was from 16.8% to 31.4% in studies that did not evaluate previous cognitive status.^24-33^ In a population-based study of prevalence of early dementia after first-ever stroke, 3201 patients were evaluated, of which 20.4% had post-stroke dementia.^34^ In a study conducted in Chile, evaluation one year post-stroke revealed 39% cognitive impairment no dementia and 22% dementia.^35^

In a systematic literature search by Snaphaan & de Leeuw, the prevalence of post-stroke memory dysfunction differed depending on the follow-up period, varying from 23-55% three months after stroke to 11-31% one year after stroke.^36^

Our aims were to evaluate the prevalence of VCI in an ischemic stroke cohort over a 12-month follow-up period, and to verify risk factors involved in the development of VCI in patients of non-embolic stroke.

## METHODS

We evaluated 172 consecutive stroke patients who were followed for a 12-month period. The inclusion criteria were: age ≥55 years of age; transitory ischemic attack (TIA), atherothrombotic ischemic stroke (small or big vessels) (TOAST 1 and 3), not submitted to thrombolysis. We excluded embolic strokes, except for patients with contraindication for oral anticoagulation (TOAST 5) and patients with severe disability, and who were bedridden. All patients had undergone computed tomography or magnetic resonance imaging. The first evaluation was performed 30±20 days after stroke, and the second assessment was done at 12 months post-stroke (±10 days).

On the first visit (V1), patients were evaluated according to demographic data (gender, age, schooling), risk factors (smoking, systemic arterial hypertension - SAH, diabetes mellitus-DM, cholesterol, triglycerides, uric acid, and body mass index-BMI), and TOAST classification (TOAST-Trial of Org 10172 in Acute Stroke Treatment).^37^

Brief cognitive screening was performed with the Mini-Mental State Examination-MMSE,^38^ and physical impairment assessed by the Modified Rankin Scale (MRS).^39^

On the second visit (V12), patients were evaluated with the MMSE, MRS and also for stroke recurrence and death. All patients with MMSE scores of less than one standard deviation from the mean for schooling level were referred for cognitive evaluation; according to Brucki et al. (2003): illiterates - <17 points; 1 to 4 years of schooling <21 points; 5 to 8 years <24 points; and 9 or more years of education <26 points.

After cognitive and functional evaluation, patients were classified into without cognitive impairment (WCI); cognitive impairment no dementia (CIND) - patients with cognitive impairment in one or more cognitive domains (executive function, attention, memory, visuospatial function, language) but with preserved functional activities (<5 points on Functional Activities Questionnaire);^40^ and vascular dementia according to NINDS-AIREN criteria.^41^ Classification was based on clinical impression after neurological and cognitive evaluation (adapted for each patient) by the same examiner (SMDB).

All patients signed an informed consent form. The study was approved by the Research Ethics Committee of Hospital Santa Marcelina. Statistical analyses were performed using SPSS 17.0; non-parametrical tests were performed for analysis of demographic data, and multiple regressions were performed to evaluate possible risks associated to cognitive impairment in stroke patients.

## RESULTS

Patients comprised 81 women (47.1%) and had a mean age of 67.77 (7.86) years, schooling of 3.52 (2.99) years, and MMSE score of 24.94 (3.59) points.

The sample was predominantly young: 20.9% from 55 to 60 years of age, 43% from 61 to 70 years, 29.1% between 71 and 80 years, and only 7% were older than 80 years of age. Schooling level was very low with 22.1% illiterates and 59.3% of patients with 1 to 4 years of education.

Patients were classified by TOAST into: 55.8% as TOAST 1 (n=96); 28.5% (49 patients) as TOAST 3 (n=49), and 9.3% as TOAST 5 (n=16); nine patients had TIA.

Vascular risk factors were assessed in these stroke patients as follows: SAH in 93% patients, current smoking in 46.5%, DM in 37.8%, elevated triglycerides in 30.8%, elevated cholesterol in 25.6%, and elevated uric acid in 18%. BMI was distributed as: eutrophic in 40.7% (BMI: 18.5-24.9); overweight in 46.5% (BMI: 25-29); and mild obesity in 9.9% (BMI: 30-34.9).

During the 12-month period only one stroke was observed in 90.4% of patients (n=150), 15 patients (9.4%) had a second stroke whereas one patient had a third stroke (0.6%). Ten patients died (5.8%) during the study. Sixteen patients did not undergo the second evaluation.

**Figure 1:**
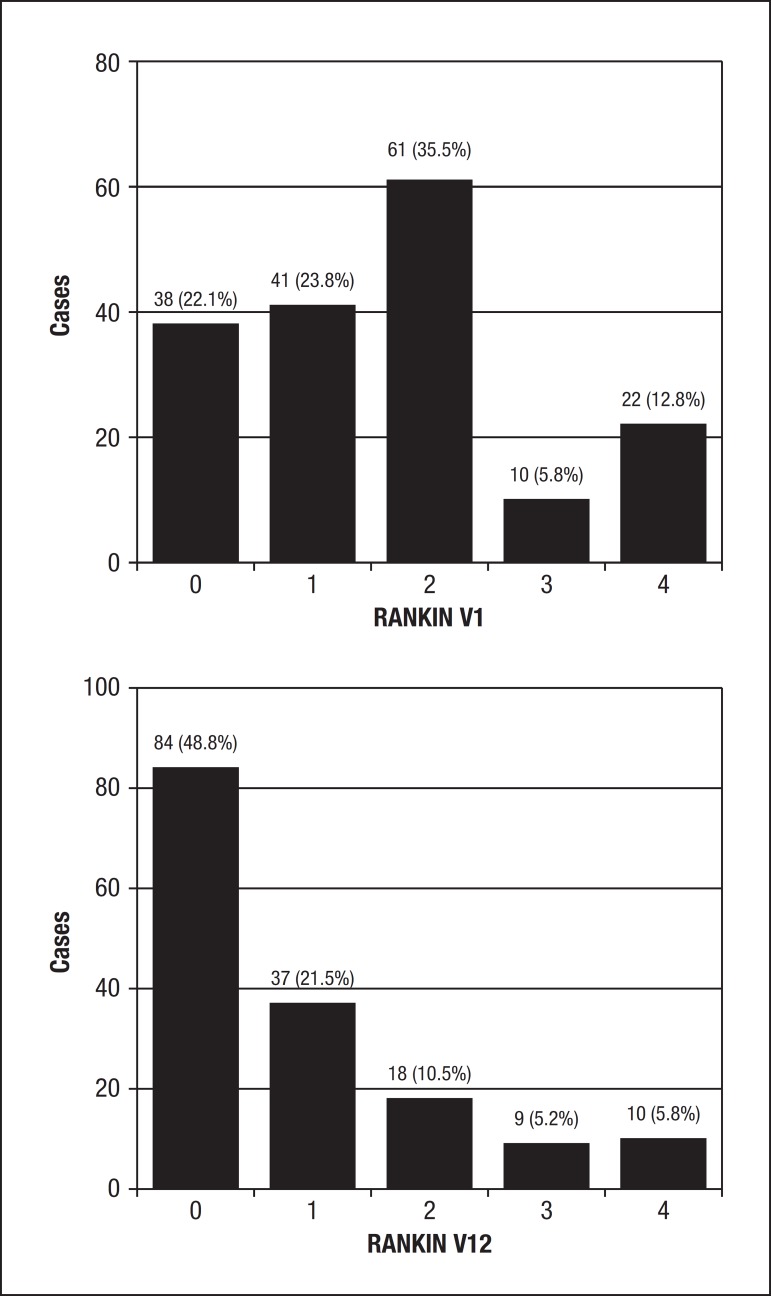
Distribution of patients by Modified Rankin scores on visit 1(left) and visit 12 (right).

The distribution by the Modified Rakin Scale is given in Figure 1. We can observe that at V1, 45.9% of the patients were classified as 0 and 1 (no symptoms and no significant disability despite symptoms; able to carry out all usual duties and activities, respectively). After one year post-stroke, a considerable proportion presented with no symptoms or only mild disability (70.3%).

On cognitive evaluation, 73.8% had a diagnosis of WCI, 4.6% were diagnosed as CIND, and 12.2% had a diagnosis of dementia (probable vascular dementia in 20 patients and one subject with cerebrovascular disease and Alzheimer's disease). Therefore, the results showed that 16.8% of our stroke sample developed VCI.

To evaluate predictors of cognitive impairment, patients with CIND and dementia were pooled into a single group. Table 2 depicts the distribution of vascular risk factors between groups without cognitive impairment and with cognitive impairment for four factors differing between groups (illiteracy, smoking, and severity of disability on visit 1 and 12). Patients diagnosed as VCI had a greater likelihood of being illiterates, nonsmokers, and less disabled (MRS of 0 to 2). Multiple regression analysis yielded the predictors of VCI as schooling, nonsmoking and severity of MRS on visit 1.

**Table 2:** Distribution of vascular risk factors and Rankin scores between WCI patients and VCI patients.

	Pearson's Chi-Square	p-value
Age	6.78	0.079
Education = 0	4.26	0.039
Smoking*	9.06	0.003
SAH*	0.001	0.971
DM*	3.226	0.072
Cholesterol*	1.981	0.159
Triglycerides*	0.001	0.973
BMI overweight/obesity	1.245	0.871
Rankin V1	10.926	0.027
Rankin V12	20.569	<0.001

* Presence of risk factor.

Twenty-eight percent of patients with VCI had MRS of 3 or 4 on V12 (moderate disability; requiring some help, but able to walk without assistance - 3; moderately severe disability; unable to walk without assistance and unable to attend to own bodily needs without assistance - 4), in contrast to patients with no cognitive impairment, 8.3% of whom had MRS of 3 or 4.

## DISCUSSION

To our knowledge, the present study is the first evaluating prevalence rates of VCI in Brazil among patients with non-embolic stroke. We followed-up patients for one year, a period similar to other studies in the pertinent literature. There is a scarcity of data on VCI with the majority of studies reporting only the prevalence of dementia and most evaluating patients with all causes of stroke. We restricted our sample to stroke of non-embolic origin to regulate risk factors to those associated with atherothrombosis. The prevalence of VCI found was 16.8%, and dementia was present in 12.2% of the 172 patients. This prevalence was among the lowest reported, but some characteristics of this investigation differed to other studies, mainly in relation to patient schooling, age, and method of evaluation.

The majority of studies have used DSM-III, DSM-IIIR, DSM-IV or ICD-10 for diagnosing vascular dementia.^22^ However, NINDS-AIREN was employed in the present study, constituting a more specific instrument for this diagnosis.^42^ Prevalence of VCI increases with age,^8^ and our sample contained 63.9% of patients aged between 55 and 70 years, representing a relatively young sample. However, our rate could be higher considering level of education, since 81.4% had less than five years of schooling, where some studies have shown educational level to be a risk factor for developing dementia after stroke. Although illiterates were more frequent among patients with VCI, illiteracy was not a major factor elevating the prevalence of cognitive decline in our population.

Our inclusion criteria were very restrictive, since the majority of patients having ischemic stroke with an atherothrombotic mechanism (TOAST 1 and 3) were included. Excluding embolic strokes.One of the risk factors was recurrence of stroke, but among our patients 90.4% had only one stroke during the follow-up period. Nevertheless, some studies analyzing the prevalence of post-stroke dementia after recurrence observed a much greater rate.^22^

The risks related to developing VCI were schooling, nonsmoking, and severity of functional state (measured by MRS). In a previous meta-analysis, significant predictors of post-stroke dementia were demographic factors (older age, low educational attainment, previous cognitive decline, premorbid disability) and vascular risk factors including diabetes and atrial fibrillation, but not hypertension, ischemic heart disease, cholesterol, previous transient ischemic attack, or previous smoking.^22^ Among our risk factors, we considered BMI, but overweight and obesity did not represent a risk for VCI.

Patients were referred for cognitive evaluation based on their MMSE scores. MMSE is not a good screening test in vascular dementia patients, particularly in subcortical vascular disease or in patients with language problems but many studies have used it to diagnose for dementia.^43,44^ In the present study however, it was used as a screening tool with subsequent cognitive evaluation performed on patients with decline on the MMSE. We are in agreement with the consensus on evaluation of vascular dementia proposed by the Brazilian Academy of Neurology, which includes this test in brief screening protocols, besides the verbal fluency test (animal category) and clock drawing test.^44^

There are some limitations in our study that should be pointed out. We evaluated patients using a comprehensive basic battery, incorporating the four main domains included in Gorelick et al. proposed criteria for VCI.^1^ In addition, we performed an evaluation of different functions according to observed impairment during the neurological evaluation, such as agnosia, apraxia, abstract reasoning. Alcoholism was not screened formally, only through a question on drinking habits, later removed from risk factor analysis. No depression scale was used to exclude depressive patients or those at risk of depression. However, the study was previously performed as the patients are normally attended in clinical settings, and many patients were in use of antidepressant medication although none fulfilled major depression criteria. Finally, we did not include imaging findings in the analysis, such as white matter disorders, lacunae and microbleeds because the study scope was to determine the general prevalence of VCI in a stroke cohort in Brazil .

In conclusion, post-stroke VCI is a very important issue due to its high prevalence, for which the main treatments are acute stroke care and secondary prevention, measures that must be included in our health care system.

## Figures and Tables

**Table 1:** Demographic data and MMSE scores on the two visits

	Mean (Sd)	Median	Range
Age	67.8 (7.9)	67	55-91
Education	3.5 (3.0)	4	0-15
MMSE V1	25.3 (3.5)	25	16-30
MMSE V12	25.2 (4.3)	26	9-30

**Table 3:** Multiple regression - risk factors and cognitive status.

		B	S.E.	Wald	Df	sig	Exp (B)
Step 1^a^	Smoking	-1.351	0.469	8.286	1	0.004	0.259
	Constant	-0.952	0.251	14.386	1	<0.001	0.386
Step 2^b^	Smoking	-1.311	0.477	7.544	1	0.006	0.270
	RankinV1	1.227	0.511	5.770	1	0.016	3.411
	Constant	-1.209	0.284	18.091	1	<0.001	0.298
Step 3^c^	Schooling	-1.080	0.498	4.701	1	0.003	0.340
	Smoking	-1.251	0.484	6.694	1	0.010	0.286
	RankinV1	1.227	0.525	5.460	1	0.381	3.412
	Constant	-0.401	0.458	0.767	1	0.381	0.670

a Variable entered in step 1: Smoking;

b Variable entered in step 2: Rankin V1;

c Variable entered in step 3: Schooling 0 and 1.
